# Lipids, blood pressure and kidney update 2015

**DOI:** 10.1186/s12944-015-0169-0

**Published:** 2015-12-30

**Authors:** Maciej Banach, Wilbert S. Aronow, Maria-Corina Serban, Jacek Rysz, Luminita Voroneanu, Adrian Covic

**Affiliations:** Department of Hypertension, Chair of Nephrology and Hypertension, Medical University of Lodz, Zeromskiego 113, 90-549 Lodz, Poland; Department of Medicine, New York Medical College, Valhalla, NY USA; Department of Epidemiology, University of Alabama at Birmingham, Birmingham, AL USA; Department of Functional Sciences, Discipline of Pathophysiology, “Victor Babes” University of Medicine and Pharmacy, Timisoara, Romania; Nephrology Clinic, Dialysis and Renal Transplant Center, C.I. Parhon University Hospital and Grigore. T. Popa, University of Medicine and Pharmacy, Iasi, Romania

**Keywords:** Blood pressure, Cholesterol, Dyslipidemia, Hypertension, Lipids, Renal disease, Statin intolerance statin myopathy

## Abstract

The most important studies and guidelines in the topics of *lipid, blood pressure and kidney* published in 2015 were reviewed. *In lipid research*, the IMProved Reduction of Outcomes: Vytorin Efficacy International Trial (IMPROVE-IT) trial revalidated the concept “lower is better” for low density lipoprotein (LDL)-cholesterol as a target for therapy, increasing the necessity of treatment the high-risk patients to achieve LDL-C goals. After these results, ezetimibe might become the preferred additional drug in the combination therapy of lipid disorders because of oral dosage form and lower acquisition cost. However, for the statin-intolerant patients and those patients requiring essential reductions in LDL-C to achieve their goals, new therapies, including PCSK9 inhibitors remain promising drugs. *In blood pressure research*, American Heart Association (AHA)/American College of Cardiology (ACC) 2015 guidelines recommended a target for blood pressure below 140/90 mmHg in stable or unstable coronary artery disease patients and below 150/90 mmHg in patients older than 80 years of age, however the recent results of the Systolic Blood Pressure Intervention Trial (SPRINT) trial have suggested that there might be significant benefits, taking into account cardiovascular risk, for hypertensive patients over 50 without diabetes and blood pressure levels <120/80. *In kidney research*, reducing the progression of chronic kidney disease and related complications such as anemia, metabolic acidosis, bone and mineral diseases, acute kidney injury and cardiovascular disease is still a goal for clinicians.

## Background: ​Lipids update 2015

The year 2015 was an important year for lipid research. The researchers were still focused on best strategies for dyslipidemia management, by using various combinations of statins with other lipid-lowering drugs or testing new therapies [1]. First, the IMProved Reduction of Outcomes: Vytorin Efficacy International Trial (IMPROVE-IT) revalidated the concept that low density lipoprotein cholesterol (LDL-C) levels are a relevant treatment goal [2, 3]. Moreover, the IMPROVE-IT trial showed that the combination with ezetimibe/simvastatin 10 mg/40 mg let to an absolute 2.0 % reduction (relative risk reduction: 6.4 %; *p* = 0.016) of the risk of cardiovascular (CV) events (a composite of CV death, myocardial infarction [MI], unstable angina requiring rehospitalization, coronary revascularization or stroke) in contrast to simvastatin 40 mg alone with 7-year number needed to treat (NNT) = 50 [2]. IMPROVE-IT trial also demonstrated that the patients with obtained very low LDL-C levels <30 mg/dl experienced no discrepancies in adverse effects (events producing discontinuation of therapy, muscle-related events, cognitive dysfunction, liver enzymes elevations, or hemorrhagic stroke) than those with higher LDL-C levels [2]. Beside IMPROVE-IT trial, another important trial ODYSSEY LONG-TERM (NCT01507831) with proprotein convertase subtilisin/kexin type 9 (PCSK9) inhibitor - alirocumab, supported the hypothesis ‘even lower is even better’ for LDL-C levels [4], generating more arguments for lower LDL-C targets <50 mg/dl (1.3 mmol/l), in contrast with the current targets <70 mg/dl (1.8 mmol/l) for patients at the highest risk [5]. The same trial also confirmed that LDL-C goals below <25 mg/dl and even <15 mg/dl were not connected with the increase of any adverse events [5, 6]. Similar results were obtained in the Open-Label Study of Long-Term Evaluation Against LDL-C (OSLER) trial with evolocumab [7]. However, the data concerning the safety of preserving very low LDL-C levels on long term are still limited [6].

## What next after approval of the PCSK9 inhibitors?

Monoclonal antibodies to PCSK-9 can reduce LDL-C levels by even more than 60 % in statin users [8–10]. Recently, two first PCSK9 inhibitors (evolocumab - Repatha, Amgen, and alirocumab – Praluent, Sanofi) have been approved worldwide by both Food and Drug Administration (FDA) as well as European Medicine Agency (EMA) for the treatment of uncontrolled LDL-C levels in high risk patients [10, 11]. According to FDA and EMA, PCSK9 inhibitors were approved to treat adult patients with primary hypercholesterolemia (heterozygous familial hypercholesterolemia [HeFH] and non-familial) or mixed dyslipidemia as an adjunct to diet: a) in patients unable to reach their LDL-C goals with a maximally-tolerated statin, as a combination therapy with a statin, with or without other lipid-lowering therapies; and b) for patients who are statin intolerant, or for whom a statin is contraindicated, PCSK9 inhibitors would be used alone or in combination with other lipid-lowering therapies (http://www.ema.europa.eu/ema/index.jsp?curl=pages/news_and_events/news/2015/07/news_detail_002377.jsp&mid=WC0b01ac058004d5c1).

The safety and tolerability of evolocumab was tested in abovementioned OSLER trial on hypercholesterolaemic patients [12]. Indeed, evolocumab reduced the levels of LDL-C by 61 % (*p* <0.001), what was associated with the significant reduction of CV events (however the study was not designed to analyze this effect). The researchers also noticed that neurocognitive events were more common in the evolocumab *vs* standard therapy group (0.9 *vs* 0.3 %) [13]. The safety and efficacy of alirocumab was tested in a randomized trial on 2341 statin users (ODYSSEY LONG-TERM) with LDL-C levels ~ 70 mg per deciliter (1.8 mmol per liter) with high CV risk as compared with the placebo group [14]. The researchers showed a significant reduction of LDL-C (−62 % from baseline) with alirocumab as compared to placebo group (*p* <0.001) after 24 weeks of therapy [14]. The patients in the alirocumab group were also observed to have increased risk of neurocognitive events (1.2 % *vs* 0.5 %), myalgia (5.4 % *vs* 2.9 %), ophthalmologic events (2.9 % *vs* 1.9 %) and higher rates of injection-site reactions (5.9 % *vs* 4.2 %), compared to placebo group [14]. Similarly to the results in the OSLER trial, the rate of CV events was significantly decreased in alirocumab than in placebo group (1.7 % *vs* 3.3 %, *p* = 0.02, however again the study was not designed to analyze these outcomes) [14]. The efficacy and safety of alirocumab and evolocumab on major CVD events at 5 years is still under evaluation in ODYSSEY OUTCOMES (Evaluation of Cardiovascular Outcomes after an Acute Coronary Syndrome during Treatment with Alirocumab; NCT01663402) [15] and FOURIER (Further Cardiovascular Outcomes Research with PCSK9 Inhibition in Subjects with Elevated Risk; NCT01764633) [16] trials. The results will be available in 2017. The third PCSK9 inhibitor – bococizumab (Pfizer) has been also under evaluation, and two trials – the Studies of PCSK9 Inhibition and the Reduction of vascular Events-1 (SPIRE-1) and SPIRE-2 are ongoing, and the first results will be also available in 2017 (https://clinicaltrials.gov/ct2/show/NCT01975376). The issue of potentially increased risk of neurocognitive disorders is a matter of further investigations (and their association with very low levels of LDL-C), especially after publication of recent meta-analysis, suggesting significant increase of these PCSK9 possibly related side effects (https://clinicaltrials.gov/ct2/show/NCT01975389?term=SPIRE-2&rank=1). There is also specially designed study (ongoing) – the Evaluating PCSK9 Binding antiBody Influence oN coGnitive HeAlth in High cardiovascUlar Risk Subjects (EBBINGHAUS) - to analyze the effect of evolocumab therapy on the risk of neurocognitive disorders (https://clinicaltrials.gov/ct2/show/NCT02207634?term=pcsk9+neurocognitive&rank=1).

In 2015 there have been a number of new studies and analyses evaluating of PCSK9 inhibitors effect of different parameters of lipid profile. The effects of alirocumab 150 mg every 2 weeks on lipoprotein a [Lp(a)] levels in hypercholesterolemic on lipid-lowering drugs were evaluated in pooled data from three double-blind, randomized, placebo-controlled, phase 2 studies (NCT01266876, NCT01288469, and NCT01288443), after 8–12 weeks of therapy [17]. This study was an effect of large discussion on the real effect of PCSK9 inhibitors on Lp(a) – independent risk factor of CVD [8, 18, 19]– as the previous studies suggested that the effect might have been related to the baseline Lp(a) values [8, 9]. The levels of Lp(a) were significantly reduced in alirocumab group compared with placebo (−30.3 % *vs* −0.3 %, *p* <0.0001) [17].

The effect of alirocumab 150 mg every 2 weeks on lipoprotein particle size and concentration in hypercholesterolemic patients (LDL-C levels ≥100 mg/dL) on a stable atorvastatin dose was recently tested using nuclear magnetic resonance spectroscopy in a phase II, double-blind, placebo-controlled trial [20]. The mean concentrations of total very-low-density lipoprotein particle concentrations (−36.4 % *vs* +33.4 %), small (−54.0 % *vs* +17.8 %), large (−71.3 % *vs* −21.8 %) and total LDL-P (−63.3 % *vs* −1.0 %) subfractions were significantly reduced after alirocumab therapy *vs* placebo (all *p* <0.01). On the contrary, it was noticed a higher increase of large (+44.6 %) *vs* medium (+17.7 %) and small HDL-C particles (+2.8 %) and total HDL-C particles (+11.2 % *vs* +1.4 %, *p* <0.01). in alirocumab group as compared to placebo group [20]. These results are very important taking into account the continuing discussion on the role of different subfractions/subpopulations on LDL-C and HDL-C (as well so-called dysfunctional HDL) on the progression of atherosclerosis [21–25].

A Meta-Analysis of 20 Randomized Controlled Trials (RCTs) (*n* = 9880 patients) evaluated the efficiency and safety of PCSK9 inhibitors on hypercholesterolemia [26]. It has been shown that PCSK9 inhibitors significantly decreased LDL-C, total cholesterol, triglycerides, apolipoprotein-B and Lp(a) levels and increased HDL-C and apolipoprotein-A1 levels [26]. No significant difference in terms of the discontinuation of treatment and treatment-emergent adverse events between the two groups was observed [26]. Another meta-analysis of 24 RCTs (*n* = 10 159 patients) compared the effects of PCSK9 inhibitors *vs* placebo on lipid and CV events [27]. The levels of LDL-C, total cholesterol, and Lp(a) were significantly reduced (48, 31 and 26.5 %, respectively, all *p* <0.001), while the level of HLD-C was significantly increased (6 %, *p* <0.001) in PCSK9 inhibitors *vs* placebo groups [27]. Despite the fact that the number of CV events was very small, the authors showed reduced rate of MI with use of PCSK9 antibodies (odds ratio [OR] 049, 95 % Cl: 0.26 to 0.93; *p* = 0.030), all-cause mortality (OR 0.45, 95 % CI: 0.23 to 0.86; *p* = 0.015), and CV mortality (OR 0.50, 95 % CI: 0.23 to 1.10; *p* = 0.084 [27]. Consecutively, another meta-analysis of 25 randomized controlled trials (*n* = 12 200 patients) tested the safety and efficacy of alirocumab and evolocumab as compared with placebo in terms of the discontinuation of treatment and treatment-emergent adverse events [28]. The risk of injection-site reactions was increased after alirocumab (RR = 1.48; 95 % CI = 1.05 to 2.09), but not after evolocumab therapy (RR = 1.06; 95 % CI = 0.67 to 1.67), as compared with placebo [28]. As compared with control, alirocumab decreased the rates of death and abnormal kidney function, whereas evolocumab decreased the rate of abnormal liver function (RR = 0.43; 95 % CI = 0.20 to 0.93) [28]. Alirocumab also reduced the risk of MI by 51 % (*p* = 0.03) and of all-cause mortality by 55 % (*p* = 0.015) [28].

A recent retrospective, cross-sectional, observational study evaluated the clinical characteristics of 164 PCSK9 gain of function (GOF) mutation carriers [29]. It was noticed that 16 different PCSK9 GOF mutations were related to various severe LDL-C levels [29]. Than the PCSK9 GOF mutation carriers were randomized to be treated during 8 weeks with either alirocumab or placebo [29]. After 2 weeks of treatment it was noticed a significant decrease - 62.5 % *vs* 53.7 % (*p* <0.0001) of LDL-C levels and after 8 weeks of treatment the observed reduction was even 73 % (*p* <0.0001) of LDL-C levels in alirocumab *vs* placebo group [29]. It is very important study, indicating large effectiveness of PCSK9 inhibitors even in the patients with highest CV risk with genetic predisposition. Another PCSK9 inhibitor, bococizumab, has been recently tested for safety in experimental studies on pregnant Sprague-Dawley (SD) rats [30]. The maternal, fetal exposure, tolerability and pharmacodynamic effects and definitive embryo-fetal development toxicity following maternal administration of bococizumab were evaluated [30]. The results indicated no embryo-fetal toxicity of bococizumab administration in pregnant rats, revalidating the rats as proper models for the safety evaluation [30]. This important study as the first indicates that PCSK9 inhibitors might be effective and especially safe as a potential lipid lowering therapy in pregnant women group, where we have very limited options to treat dyslipidemia effectively [31–33].

Bococizumab has been also observed to be efficacious and safe at a dose 150 mg every 2 weeks in a phase II clinical trial on 354 hypercholesterolemic statin users (LDL-C ≥80 mg/dL) [34]. After 12 weeks, the most effective bococizumab doses to decrease LDL-C levels were the 150 mg every 2 weeks (−53 mg/dl) and the 300 mg dose every 4 weeks (−45 mg/dL) [34]. Furthermore, the safety and efficacy of bococizumab 150 mg every 2 weeks is currently tested in high risk patients for cardiovascular events in two placebo-controlled phase 3 trials, SPIRE-1 (*n* = 17 000 patients with LDL-C levels =70–100 mg/dl) [35] and SPIRE-2 (*n* = 9000 patients with LDL-C levels ≥100 mg/dl) [36]. The final data will be available for SPIRE-1 trial in June 2018 and for SPIRE-2 trial in March 2018.

## Statin intolerance – the discussion goes further

The International Lipid Expert Panel (ILEP) recently recommended a unified definition for statin intolerance [37, 38] supplementing the EAS statement on statin associated muscle symptoms (SAMS) [39]. The National Lipid Association’s Muscle Safety Expert Panel proposed a myalgia clinical index score [40]. Taking all these into account, it seems, that definition of statin intolerance - *the inability to tolerate a dose of statin required reducing a person’s CV risk sufficiently –* is the easiest to understand not only by the specialists but especially by general practitioners. The discussion around statin intolerance/statin induced myopathy (SIM)/statin associated myopathy is mainly connected to the fact that the lipidologists face the challenge of large discontinuation of statin therapy- even 75 % within 2 years, accusing in about 60 % of cases statin-associated muscle symptoms [41]. Therefore, the awareness of different statin therapy-related side effects might result in effective prevention of this unfavorable phenomenon, fast diagnosis and implementation of suitable management [37, 38].

Besides muscle symptoms in statin intolerant patients, various statin-side effects such as sleep problems, hair loss, joint pains, pseudo-lupus syndrome, gastroenterological disorders, peripheral neuropathy, sexual function problems, weight change, have been described [42–44]. The main problem however is associated to the fact that we have not had enough data in order to confirm the causality of statin therapy and the occurrence of these symptoms. A recent systematic review and meta-analysis of five trials comprising nine treatment arms showed that statins did not modified the duration and efficacy of sleep, entry to stage I, and latency to stage I sleep, but significantly decreased the number of awakenings and wake time [45].

Today, the main challenge associated with statin therapy in the context of statin intolerance, after elimination of various causes of muscle symptoms (as well as *nocebo effect* phenomenon), is discontinuation of statin therapy (or reduction of dose) and then re-administration (rechallenge) of very low dose of statins and slow consecutive up titration, until the maximally tolerated dose is established [46]. It is important to emphasize that using “*take your time*” approach we are able to keep the statin therapy in even 90 % of patients previously suggested as statin intolerant. Taking into account the above definition it does not mean these patients are not statin intolerant (=partial intolerance), however, it is important to emphasize that having and opportunity to use even small dose of statin, we are able to prevent CV risk much more effectively [47–49].

Some potential mechanisms of statin-induced myopathy have been described, including raised atrogin-1 expression, reduced dolichols or decreased cholesterol synthesis and production of prenylated proteins [50]. Recently, it has been suggested that statin-induced myopathy might be related to mitochondrial complex III inhibition, identifying a new target for therapy [51]. More recent studies showed that the carriers for carnitine palmitoyltransferase II deficiency and McArdle disease have increased prevalence of statin-induced myopathy [52]. The research of statin intolerance phenomenon was also concentrated on gene variants involved in metabolic pathways, such as deficiency of coenzyme Q, isoprenoid production or fatty acid oxidation [53]. Additionally, the variants of genes coding for the cytochrome P450 (CYP) enzyme superfamily were intensively studied [54]. A recent genome-wide association study showed that the ‘G’ minor allele of rs13064411 in the WD repeat domain 52 gene is linked to LDL-C reaction to statins and statin-induced PCSK9 levels [55], whereas the same minor allele of this single-nucleotide polymorphism was associated with decreased LDL-C lowering response to statins [56]. Additionally, the minor allele of exon 12 of the LDL receptor (LDLR) gene, situated in the β-propeller region, changes the LDL-R intracellular distribution in a hepatoma cell line [57]. Moreover, a comprehensive comparison of LDLR variants upon ethnicity was recently performed [58]. It has been revealed that rs1003723, rs5925, rs688, rs1799898, rs6413504 were more prevalent in Europeans, rs14158 in Asians and rs11669576 in Africans [58]. Therefore it seems that not only statin intolerance but also lipid lowering drug response and LDL-C reduction might be genetically determined.

Different strategies have been tried for the management of statin intolerance phenomena. First it needs to emphasize that there is common agreement that before therapy implementation of statin intolerance, we should consider always asking for family history of statin-related side effects, about physical exertions, as well as checking the level of thyroid hormones and vitamin D concentration. Some also consider CoQ10 concentration measurement [9, 37, 38]. Since statins obstruct the coenzyme Q10 (CoQ10) production, therefore the hypothesis of CoQ10 deficiency involvement in statin-associated myopathy quickly appeared [59]. A systematic review and meta-analysis of nine randomized controlled trials (*n* = 302 patients) showed no effects of CoQ10 supplementation on statin-induced myopathy, however it needs to be emphasized that CoQ10 was administered at doses up to 400 mg/day only [60]. The update of this meta-analysis with CoQ10 supplementation doses up to 600 mg/day showed, however, the same negative results [61]. Another factor potentially susceptible for involvement in statin-induced myopathy is the deficiency of vitamin D levels [62]. Indeed, a systematic review and meta-analysis of seven randomized controlled trials (*n* = 2420 patients) showed that low vitamin D levels are associated with myalgia in statin users [63]. Recent cross-sectional study on 5907 participants ≥40 years old proved that statin users with 25(OH)D <15 ng/mL had 1.9 times higher odds of myopathy compared to non-statin users [64]. Another study examined the relation between risk of statin-induced myopathy and genetic polymorphism in the vitamin D receptor (VDR) [65]. The study showed a four times greater risk of myopathy in individuals homozygous for the C allele in the VDR polymorphism *TaqI* (rs731236) (RR 4.37, 95 % CI: 1.9–10.1, *p* <0.01) and in patients with 25OHD levels <50 nmol/L (RR 4.2; 95 % CI: 1.7–10.2; *p* <0.01) [65].

PCSK9 inhibitors – evolocumab and alirocumab - seem to be the safe and very effective alternative for the high risk patients with statin intolerance [66, 67], however bococizumab is still under development to treat statin intolerance (NCT Trial Identifier: NCT02135029). Another option is ezetimibe, especially now, when we have strong CV data supporting its efficacy and safety [6, 68]. The results of IMPROVE-IT trial reconsidered ezetimibe as a potent LDL-C lowering agent for patients after acute coronary syndrome, including statin intolerant patients, and revalidated the concept “*even* lower is *even* better” for LDL-C levels [6, 69]. Indeed, the trial showed that ezetimibe/simvastatin combination led to an absolute 2.0 % reduction (relative risk reduction: 6.4 %) of the risk of CV events *vs* simvastatin alone, at 7 years with [69]. The results at only 5.5 years of the IMPROVE-IT trial showed no significant increase in new-onset diabetes in patients on ezetimibe/simvastatin combination *vs* simvastatin only users [69]. The implications of IMPROVE-IT trial have been recently evaluated in a large health care system [70]. The study showed that 69,508 (31.6 %) of the 219,625 patients with acute coronary syndrome, might be ideal candidates for ezetimibe therapy based on the following criteria: LDL-C = 50–125 mg/dL and no use of statins more potent than simvastatin 40 mg. However, new LDL-C lowering drugs as an add-on therapy to statins or in place of statins in statin-intolerant patients are still under development, because of the modest efficacy of ezetimibe (about additional 15–20 % LDL-C reduction) [71].

## HDL-C remains a target for therapy?

It is already known that the most important mechanism by which HDL-C exerts its anti-atherogenic effects is the reverse cholesterol transport (RCT) [72]. Since cholesteryl ester transfer protein (CETP) is a key regulator of RCT, potential drugs to inhibit CETP for increasing HDL-C levels quickly appeared [73]. Torcetrapib, the first CETP inhibitors developed and tested in clinical trials, decreased by 25 % the levels of LDL-C and increased by 70 % the levels of HDL-C [74]. The development of Torcetrapib was soon after interrupted after observing an increase rate of the CV and non-CV mortality (due to significantly elevated blood pressure [BP] as a result of increased synthesis of endothelin-1 and aldosterone) [74]. Anacetrapib and dalcetrapib were the next CETP inhibitors developed, with a good efficacy and tolerability in phase I and II clinical trials [75]. Data and Safety Monitoring Board (DSMB) soon recommended the interruption of the development of dalcetrapib upon no clinically efficacy noticed in phase III clinical trials [76]. The efficacy of anacetrapib as LDL-C lowering agent was evaluated in the Study to Assess the Tolerability and Efficacy of Anacetrapib Co-administered With Statin in Participants With Heterozygous Familial Hypercholesterolemia (REALIZE) trial (*n* = 204 patients) - a randomised, double-blind, placebo-controlled, phase 3 study [77]. At week 52, anacetrapib decreased by 36 % the levels of LDL-C compared with increased levels LDL-C by 3.7 % with placebo [77]. The patients on anacetrapib had a slight increase of CV events compared to placebo (2 % vs. 0 %, *p* = 0.1544), but a similar number of adverse events resulting in discontinuation (6 % *vs* 5 %) [77]. The effects of anacetrapib 100 mg daily *vs* placebo on plasma lipids at 24 weeks was evaluated in the Determining the Efficacy and Tolerability of CETP INhibition with AnacEtrapib (DEFINE) trial [78]. The percent decreases in Friedewald calculation (Fc)-LDL-C and increases in HDL-C with anacetrapib were similar subgroups by age, gender, diabetes status, lipid-modifying regimen, and baseline Fc-LDL-C, HDL-C, or TG. On the other hand, anacetrapib effects on Fc-LDL-C (−24 % *vs* −41 %) and HDL-C (+75 % *vs* +139 %) appeared to be less in black vs white patients, respectively [78]. The clinical impact of the lipid-modifying effects of anacetrapib on CVD outcome (*n* = 30 624 patients) will be reported in 2017, when the Randomized EValuation of the Effects of Anacetrapib through Lipid-modification (REVEAL) trial will be finished (https://clinicaltrials.gov/ct2/show/NCT01252953). However, the long half-life of anacetrapid, with plasma levels still noticeable after 2–4 years after the last dose, might cause some problems in any attempt for the approval of this drug. On the other hand, it seems that anacetrapib is the most potent CETP inhibitor taking into account HDL-C increase (even +140 %) and reduction of LDL-C (even −40 %), therefore there is a chance that REVEAL results might be positive. Evacetrapib, another CETP inhibitor, was tested in A Study of Evacetrapib in High-Risk Vascular Disease (ACCELERATE) trial (NCT01687998), a phase 3, multicenter, randomized, double-blind, placebo-controlled trial (*n* = 12,092 patients) [79]. The trial was interrupted in October 2015 since an insufficient efficacy in obtaining the primary endpoint (the first occurrence in the composite cardiovascular events of cardiovascular death, myocardial infarction, stroke, coronary revascularization, or hospitalization for unstable angina) was noticed. However the safety arm of this study has been still being continued.

Until now, the therapies that modulate HDL-cholesterol levels have failed to reduce outcomes, leading to a more intense research of HDL functionality [80–82]. It is well known that the cholesterol is removed from macrophages within the arterial walls back into the bloodstream and out to the liver through the cholesterol-efflux pathway [83, 84]. Therefore, it was assumed that measuring cholesterol-efflux pathway might be a more reliable marker for CVD, compared with plasma HDL-C levels [85].

## Selected new drugs in the phase of development

### RVX-208

RVX-208 (RVX-000222) is a novel BET bromodomain antagonist and a small-molecule stimulator of apolipoprotein (Apo)-AI gene expression, developed by Resverlogix Corp (USA) for coronary artery disease (CAD) treatment [86]. Experimental studies reported that RVX-208 is capable of increasing plasma Apo A-I levels and pre-beta-HDL particles through RCT [87]. The ApoA1 Synthesis Stimulation and Intravascular Ultrasound for Coronary Atheroma Regression Evaluation (ASSURE) is a new phase 2b double blind, randomized, multicenter trial (*n* = 323 patients) that used intravascular ultrasound (IVUS) technology to evaluate the effects of RVX-208 *vs* placebo on atherosclerosis progression during 26 weeks [88]. The patients on RVX-208 had increased plasma levels of Apo A-I (12.8 % *vs* 10.6 %, *p* =0.18) and HDL-C (11.1 % vs 9.1 %, *p* =0.24) and decreased plasma levels of LDL-C (15.8 % *vs* 17.9 %, *p* = 0.55) as compared to placebo [88]. Moreover, RVX-208 decreased the change in percent atheroma volume (0.40 % *vs* 0.30 %, *p* = 0.81) and total atheroma volume (4.2 mm^3^*vs* 3.8 mm^3^, *p* = 0.86), but abnormally increased liver enzymes (7.1 *vs* 0 %, *p* = 0.009). The other study - The Study of Quantitative Serial Trends in Lipids with Apolipoprotein A-I Stimulation (SUSTAIN, NCT01423188) (https://clinicaltrials.gov/ct2/show/NCT01423188) aimed to assess the lipid efficacy, safety and tolerability of RVX-208. 176 patients with low levels of HDL-C were randomized to receive RVX-208 100 mg bid (*n* = 88) or placebo (*n* = 88) for 24 weeks. The primary efficacy parameter was the percentage change in HDL-C levels that has been met successfully (*p* <0.0001). Also, the secondary endpoints, increased apoA-I (*p* <0.0004), total and large HDL particles (*p* <0.004 for both) as well as reduced hsCRP levels at 12 weeks (*p* <0.003) were observed [87]. Taking into account some opposite results larger trials are still needed to confirm atheroprotective potential of novel HDL-targeted therapy with RVX-208, as well as its safety [87].

### ETC-1002

ETC-1002 is another new very potent LDL-C lowering drug developed by Esperion Therapeutics [89]. This drug has beneficial effects on proatherogenic lipids through enhance of the fatty acid oxidation and inhibition of fatty acid and sterol synthesis in experimental models [90, 91]. A single-center, double blind, placebo-controlled trial (*n* = 60 patients) assessed the efficacy and safety of ETC-1002 in patients with type 2 diabetes mellitus [92]. The levels of both LDL-C (43 ± 2.6 % *vs* 4 ± 2.5 %, *p* <0.0001) and high-sensitivity C-reactive protein were decreased (44 % *vs* 11 %, *p* = 0.0011) in ETC-1002 group compared with placebo group at 29 days after treatment [92]. Another multicenter, randomized, double blind, placebo-controlled trial (*n* = 177 patients) showed the efficacy (significantly decreased by 27 % the levels of LDL-C across a broad range of baseline triglycerides) and safety of ETC-1002 compared to placebo [93]. A new multicenter, double-blind, 8-week trial (*n* = 56 patients) evaluated the efficacy and safety of ETC-1002 in statin intolerant patients [94]. ETC-1002 decreased by 28.7 % the plasma levels of LDL-C more than placebo (95 % confidence interval, −35.4 to −22.1; *p* <0.0001) [94]. Furthermore, it has been shown that ETC-1002 has a similar safety and tolerability, but a greater capacity of LDL-C lowering as ezetimibe [95].

## Background: ​Blood pressure update 2015

The American Heart Association/American Society of Cardiology/American Society of Hypertension 2015 guidelines on treatment of hypertension in patients with coronary artery disease recommend that the target BP should be below 140/90 mmHg in patients with stable coronary artery disease (CAD) and with an acute coronary syndrome (ACS) if they are aged 80 years and younger and the target BP below 150/90 mmHg if they are older than 80 years of age [96–99]. Consideration can be given to lower the BP to below 130/80 mmHg with a class IIb C indication [96–99]. Octogenarians should be checked for orthostatic changes with standing and a systolic blood pressure (SBP) below 130 mmHg and a diastolic blood pressure (DBP) below 65 mmHg should be avoided [96–99]. Caution is advised in reducing DBP below 60 mmHg in patients with diabetes mellitus (DM) or in patients older than 60 years of age [96–99].

## Antihypertensive therapy

### Coronary artery disease

Coronary risk factors should be treated including cessation of smoking and treatment of hypertension, dyslipidemia, DM, obesity, and physical inactivity [96–99]. Dietary sodium should be reduced [100, 101].

Beta-blockers are the initial antihypertensive drugs to use in CAD patients who have angina pectoris, who have had a myocardial infarction (MI), and in those who have left ventricular systolic dysfunction unless contraindicated [96–99, 102, 103]. Patients with prior MI and hypertension should be treated with beta-blockers and angiotensin-converting enzyme (ACE) inhibitors [96–99, 102–104]. If a third drug is needed, aldosterone antagonists may be administered [96–99, 105]. Patients treated with aldosterone antagonists should not have significant renal dysfunction or hyperkalemia.

In addition to the beta blockers carvedilol, metoprolol CR/XL, and bisoprolol, [96–99, 106] patients with hypertension, coronary artery disease, and congestive heart failure should be treated with diuretics and ACE inhibitors or angiotensin receptor blockers (ARBs [96–99, 106], and patients with persistent severe symptoms with aldosterone antagonists [96–99, 105–107]. Hydralazine plus isosorbide dinitrate should be added to African-American patients with New York Heart Association class III or IV heart failure with a reduced left ventricular ejection fraction (HFrEF) already receiving diuretics, beta blockers, and an ACE inhibitor or ARB [96–99, 106, 108, 109]. Drugs to avoid in patients with hypertension and HFrEF include verapamil, diltiazem, doxazosin, clonidine, moxonidine, hydralazine without a nitrate, and nonsteroidal anti-inflammatory drugs [96–99].

In patients with hypertension, CAD, and heart failure with a preserved left ventricular ejection fraction (HFpEF), class I therapeutic indications include control of systolic and diastolic hypertension, control of the ventricular rate in patients with atrial fibrillation, and treatment of pulmonary congestion and peripheral edema with diuretics [96–99, 106]. Class IIb therapeutic indications include use of beta-blockers, ACE inhibitors or ARBs, or calcium channel blockers (CCBs) [96–99, 106].

### Stable angina pectoris

Patients with hypertension and chronic stable angina pectoris should be treated with beta-blockers plus nitrates as antianginal agents [96–99]. Hypertension in these patients should be treated with beta-blockers plus an ACE inhibitor or ARB with addition of a thiazide or thiazide-like diuretic if needed. If either the angina pectoris or the hypertension remains uncontrolled, a long-acting dihydropyridine CCB can be added to the therapeutic regimen. Nondihydropyridine calcium channel blockers such as verapamil and diltiazem cannot be used if there is left ventricular systolic dysfunction. Combining a beta-blocker with either verapamil or diltiazem must be used cautiously because of the increased risk of bradyarrhythmias and heart failure developing [96–99].

### Acute coronary syndromes

In patients with an ACS, initial management of hypertension should include a short-acting beta1 selective beta-blocker without intrinsic sympathomimetic activity such as metoprolol tartrate or bisoprolol [96–99]. Treatment with beta-blockers should be started initially within 24 h of symptoms. In patients with severe hypertension or ongoing ischemia, intravenous esmolol may be used [96–99]. In hemodynamically unstable patients or those with decompensated heart failure, administration of beta-blockers should be delayed until the patient is stabilized [96–99].

In patients with an ACS with hypertension, nitrates can be used to reduce blood pressure or to reduce ongoing myocardial ischemia or pulmonary congestion [96–99]. However, nitrates should not be administered to patients with suspected right ventricular infarction or in those with hemodynamic instability. Intravenous or sublingual nitroglycerin is preferred initially [96–99].

An ACE inhibitor or ARB should be given to patients with an ACS [110], especially in patients with an anterior myocardial infarction if hypertension persists, if there is an decreased left ventricular ejection fraction (LVEF), or if diabetes mellitus is present [96–99, 111]. If hypertension persists after use of a beta-blocker plus an ACE inhibitor or ARB, a long-acting dihydropyridine CCB may be added to the therapeutic regimen [96–99]. Aldosterone antagonists are indicated in patients receiving beta-blockers plus ACE inhibitors or ARBs after myocardial infarction who have left ventricular systolic dysfunction and either heart failure or diabetes mellitus [96–99]. However, aldosterone antagonists should not be given if the serum potassium is ≥5.0 mEq/L or if the serum creatinine is ≥2.5 mg/dL in men or ≥2.0 mg/dL in women [96–99]. Loop diuretics are preferred to thiazide and thiazide-type diuretics in patients with heart failure or in patients with chronic kidney disease and an estimated glomerular filtration rate less than 30 mL/min [96–99].

## Sprint

The American College of Cardiology/American Heart Association 2016 guidelines for the management of patients with hypertension will be strongly influenced by the results from the Systolic Blood Pressure Intervention Trial (SPRINT) [112]. SPRINT randomized 9,361 patients with a SBP of 130–180 mm Hg and an increased cardiovascular risk, but without diabetes mellitus, history of stroke, symptomatic heart failure within the past 6 months, a LVEF <35 %, and an estimated glomerular filtration rate <20 ml/min/1.73 m^2^ to a SBP target of <120 mm Hg or to a SBP target of <140 mm Hg [112]. The patients were aged 50 years and older with a mean age of 67.9 years. Of the 9,361 patients, 2,636 (28.2 %) were aged 75 years and older, 3332 (35.6 %) were women, 5,399 (57.7 %) were non-Hispanic white, 2,947 (31.5 %) were black, and 984 (10.6 %) were Hispanic. Cardiovascular disease was present in 1,877 patients (20.1 %), and the Framingham 10-year cardiovascular disease risk score was ≥15 % in 5, 737 patients (61.3 %).

Blood pressure was measured by use of an automated measurement system (Model 907, Omron Healthcare). At 1 year, the mean SBP was 121.4 mm Hg in the intensive treatment group (mean number of antihypertensive drugs was 2.8) and 136.2 mm Hg in the standard treatment group (mean number of antihypertensive drugs was 1.8). The intervention was stopped early after a median follow-up of 3.26 years.

The primary composite outcome was MI, other ACS, stroke, heart failure, or death from cardiovascular causes and was reduced 25 % (95 % CI, 11 % to 36 %; *p* <0.001) by intensive blood pressure treatment [112]. All-cause mortality was reduced 27 % (95 % CI, 10 % to 40 %; *p* = 0.003) by intensive BP treatment. Heart failure was reduced 38 % (95 % CI, 16 % to 55 %; *p* = 0.002)) by intensive blood pressure treatment. Death from cardiovascular causes was reduced 43 % (95 % CI, 15 % to 62 %; *p* = 0.005) by intensive blood pressure treatment. The primary composite outcome or death was reduced 22 % (95 % CI, 10 % to 33 %; *p* <0.001) by intensive BP treatment. Intensive BP treatment insignificantly reduced MI by 17 %, caused the same incidence of other ACS, and insignificantly reduced stroke by 11 %. The composite renal outcome in patients with chronic kidney disease (CKD) at baseline was insignificantly reduced 11 % by intensive blood pressure [112].

Intensive BP treatment insignificantly reduced the primary outcome 18 % in patients with prior CKD and significantly reduced the primary outcome 30 % in patients without prior CKD [112]. Intensive BP treatment significantly reduced the primary outcome 33 % in patients aged 75 years and older and significantly reduced the primary outcome 20 % in patients aged 50–74 years. Intensive BP treatment insignificantly reduced the primary outcome 16 % in women and significantly reduced the primary outcome 28 % in men. Intensive BP treatment insignificantly reduced the primary outcome 23 % in blacks and significantly reduced the primary outcome 26 % in nonblacks. Intensive BP treatment insignificantly reduced the primary outcome 17 % in patients with prior CAD and significantly reduced the primary outcome 29 % in patients without prior CAD. Intensive BP treatment insignificantly reduced the primary outcome 17 % in patients with a SBP ≥145 mm Hg, insignificantly reduced the primary outcome 23 % in patients with a SBP of 133–144 mm Hg, and significantly reduced the primary outcome 30 % in patients with a SBP of ≤132 mm Hg [112].

Serious adverse events were similar in both treatment groups [112]. However, intensive BP treatment caused more hypotension (2.4 % versus 1.4 %, *p* =0.001), more syncope (2.3 % versus 1.7 %, *p* = 0.05), more electrolyte abnormality (3.1 % versus 2.3 %, *p* = 0.02), and more acute kidney injury or acute renal failure (4.1 % versus 2.5 %, *p* <0.001). The incidence of bradycardia, injurious falls, and orthostatic hypotension with dizziness was similar in both treatment groups [112].

The effects of intensive BP treatment on renal function, dementia, and cognitive function cannot be interpreted until analysis of these end points has been completed. Data on the association between DBP and clinical outcomes and serious adverse events also need to be reported. What are the data if the DBP is reduced below 70 mm Hg, below 65 mm Hg, and below 60 mm Hg? Since hypertension is a powerful risk factor for development of heart failure, especially HFpEF, what percent of the patients who developed heart failure in SPRINT developed HFpEF?

The American College of Cardiology/American Heart Association 2016 guidelines will have to answer on the basis of expert medical opinion many questions not answered by SPRINT. What should be the target blood pressure in diabetics? In The ACTION to Control Cardiovascular Risk in Diabetes Blood Pressure (ACCORD BP) trial, reducing the SBP to <120 mm Hg in 4,733 patients insignificantly reduced the composite primary outcome of myocardial infarction, stroke, or cardiovascular death 12 % but significantly reduced the incidence of stroke (a prespecified secondary outcome) 41 %, *p* = 0.01 [113]. The sample size was much larger in SPRINT than in ACCORD BP, and there were methodological differences between both trials [114]. A post-hoc analysis of the results from ACCORD showed that the primary cardiovascular disease outcome was 26 % lower in patients randomized to intensive blood pressure treatment and standard glycemia goals than in patients randomized to standard blood pressure treatment and standard glycemia goals [114, 115].

What should the target blood pressure be in patients with prior stroke or transient ischemic attack, in patients younger than 50 years, in patients with HFrEF, and in patients with HFpEF? Although patients in SPRINT treated with intensive blood pressure control had a 38 % significant reduction in development of heart failure, SPRINT excluded patients with recent heart failure and patients with a LVEF less than 35 %. In a propensity score analysis of 7,785 patients with mild to moderate HFrEF and HFpEF, at 5-year follow-up, a baseline SBP ≤120 mm Hg was associated with increased cardiovascular and heart failure mortalities and all-cause, cardiovascular, and heart failure hospitalizations that was independent of other baseline characteristics [116].

SPRINT did not enroll patients living in nursing homes or in assisted-living facilities? How should we treat frail elderly patients with hypertension? Data from 1,130 frail patients, mean age 88 years, living in a nursing home being treated for hypertension with two or more antihypertensive drugs showed that the SBP should not be reduced to less than 130 mm Hg [117, 118].

How should SPRINT affect the recommendations regarding office versus out of office blood pressure measurements? Should SPRINT change the definitions of normal blood pressure, prehypertension, and hypertension? What should the threshold and goals be for untreated SBP between 120 and 140 mm Hg? Finally, because of a higher incidence of hypotension, syncope, electrolyte abnormalities, and acute kidney injury or failure in patients treated to a SBP less than 120 mm Hg, these patients will require more intensive monitoring for serious adverse events with an increased cost of care [119–122].

## Background: ​Kidney updates 2015

### Glomerulopathies

New conclusive data regarding rituximab administration in idiopathic membranous nephropathy were reported [123]. 80 patients with idiopathic membranous nephropathy and persistent nephrotic syndrome treated with non-immunosuppressive antiproteinuric therapy for 6 months were randomized (1:1) to be treated with rituximab (375 mg/m^2^ day 1 and day 8) or with placebo [123]. The primary composite end point was a reduction in proteinuria of at least 50 % and an increase in serum albumin of at least 30 % [123]. Secondary end points were proteinuria, serum albumin, serum creatinine, and antibodies against the M-type phospholipase A2 receptor (PLA2R-Ab). There was a significant reduction in proteinuria but without significant differences between the two groups at 3 and 6 months. However, the serum albumin levels increased significantly in the rituximab group at 3 and 6 months compared with placebo [123]. Additionally, PLA2R antibody levels dropped dramatically by month 3 in the rituximab group and stabilized after that. The differences between the two groups were significant both at month 3 (*p* <0.001) and at month 6 (*p* <0.001). Patients were followed for up to 24 months after the discontinuation of the study regimens. During this period, rates of remission were significantly higher in the rituximab group compared to the control group (64.9 % *vs* 37.5 %; *p* = 0.02) and more patients in the rituximab group achieved a complete response (7 *vs* 1) [123].

### Diabetic nephropathy

Around 20–30 % of patients with diabetes develop evidence of nephropathy - now the leading cause of end-stage renal disease (ESRD) and dialysis in the U.S. and in Europe [124]. The classical risk factors for CKD progression in diabetic nephropathy are hyperglycemia, hypertension and dyslipidemia [125]; their rigorous management could decrease the risk for renal or cardiac complications. Recently, another risk factor has been described: urinary potassium excretion. In an observational study involving 623 Japanese type 2 diabetic patients with eGFR ≥60 ml/min/1.73 m^2^ and a follow-up period of 11 year, a higher urinary potassium excretion was associated with a slower decline of renal function and lower incidence of cardiovascular complications (myocardial infarction, angina pectoris, stroke, and peripheral vascular disease) [126]. Several possible mechanisms are incriminated by the authors: 1) a BP lowering effect of K intake - the higher quartile subgroups showed lower systolic BP than the lower quartile subgroup; 2) higher amounts of potassium-rich food items, such as fresh vegetables and fruits, recognized for their antioxidant and anti-inflammatory effects; 3) a high potassium intake is reported to increase endothelium-dependent nitric oxide production and decrease salt-induced TGF-*β* production; these favorable effects may support vascular protection against atherosclerosis, which might result in preventing renal and cardiovascular complications.

Recent guidelines regarding the management of patients with diabetes and advances CKD (stage 3b or higher) were recently published by ERBP (European Renal best Practice) [127]. The most important recommendations of this guideline are listed below:

1. *Regarding glycemic control*
  ▪ Glycated hemoglobin (HbA1C) remains the routine reference to assess longer term glycaemic control in patients with CKD stage 3b or higher;
  ▪ The authors recommend ***against*** tighter glycaemic control if this results in severe hypoglycemic episodes (1B);
  ▪ The authors recommend vigilant attempts to tighten glycaemic control with the intention to lower HbA1C when values are >8.5 % (69 mmol/mol) (1C).
  ▪ The authors recommend intense self-monitoring only to avoid hypoglycemia in patients at high risk for hypoglycemia (2D).
  ▪ Metformin is still the best choice when choosing the oral hypoglycemic drugs; the authors recommend Metformin in a dose adapted to renal function as a first line agent when lifestyle measures alone are insufficient to get HbA1C in the desired range 4 (1B).
  ▪ The authors ***also strongly recommend instructing*** patients to temporarily withdraw Metformin in conditions of pending dehydration, when undergoing contrast media investigations, or in situations with an increased risk for AKI (1C).
2. *Regarding the management of cardiovascular risk in patients with diabetes and CKD stage 3b or higher*
  ▪ The authors e recommend ***not omitting coronary angiography*** with the sole intention of avoiding potential contrast-related deterioration of kidney function in patients with diabetes and CKD stage 3b or higher (eGFR <45 mL/min) in whom a coronary angiography is indicated (1D).
  ▪ Additionally, they recommend that optimal medical treatment should be considered as preferred treatment in patients with diabetes and CKD stage 3b–5 who have stable CAD, unless there are large areas of ischemia or significant left main or proximal LAD lesions (1C); moreover, when a decision is taken to consider revascularization, ***CABG is preferred over PCI*** in patients with multivessel or complex (SYNTAX score >22) CAD (1C).
  ▪ Additionally, the authors recommend that patients with diabetes and eGFR <45 mL/min who present with an acute coronary event should be treated no differently than patients with CKD and eGFR <45 mL/min without diabetes or patients with diabetes without CKD (1D).
3. *Regarding cardiovascular protection, the authors:*
  ▪ Recommend that adults with CKD stage 3b or higher (eGFRmin <45 ml/1.73 m2 or on dialysis) and diabetes who have a cardiovascular indication (heart failure, ischaemic heart disease) be treated with an ACE-I at maximally tolerated dose (1B).
  ▪ Suggest there is insufficient evidence to justify the start of an ARB in adults with CKD stage 3b or higher (eGFRmin <45 ml/1.73 m2 or on dialysis) eGFR and diabetes who have a cardiovascular indication (heart failure, ischaemic heart disease) but intolerance for ACE-I (2B).
  ▪ Recommend not combining different classes of renin angiotensin-blocking agents (ACE-I, ARBs or direct renin inhibitors) (1A).
  ▪ Suggest starting a selective beta-blocking agent as primary prevention for sudden cardiac death in patients with DM and CKD stage 3b or higher and then continuing it when tolerated (2C). They suggest prescribing lipophilic rather than hydrophilic beta-blocking agents in patients with diabetes and CKD stage 3b or higher.
  ▪ Recommend starting a statin in patients with DM and CKD stage 3b and 4 (1B).
  ▪ Suggest a statin be considered in patients with DM and CKD stage 5 (2C).
  ▪ Recommend against starting a statin in patients with DM and CKD stage 5D (1A).
  ▪ There was no consensus in the guideline development group on whether or not statins should be stopped in patients with diabetes with CKD stage 5D.
4. *Regarding BP targets, the authors only suggest:*
  ▪ Against applying lower blood pressure targets in patients with diabetes and CKD stage 3b or higher than in the general population (2C).
  ▪ That in patients with diabetes and CKD stage 3b or higher but without proteinuria, all blood pressure lowering drugs can be used equally to lower blood pressure (2C).

## Acute kidney injury

Over the past two decades, major increases in the incidence of acute kidney injury (AKI) have been reported [128]; even a minor acute reduction in kidney function is associated with an unfavorable prognosis [129]. Early AKI detection, optimal patient risk stratification and adequate treatment may improve outcomes [130, 131]. Several biomarkers have been extensively explored in the last years, but their ability to detect and predict AKI progression was inconsistent [132–134]. Two years ago, Chawla et al. described the furosemide stress test (FST): in clinically euvolemic patients with early AKI a high dose furosemide was administrated (1 mg/kg of furosemide in naive patients or 1.5 mg/kg in those with prior exposure) and urinary output was monitored two hours after furosemide administration [135]. The ideal cut-off for predicting progressive AKI during these first 2 h was a urine volume <200 ml (100 ml/h) with a sensitivity of 87.1 % and a specificity of 84.1 % [135]. The same author, in a recently published study, compared the performance of several biomarkers for AKI (including the fractional excretion of sodium, urine and plasma NGAL, urine albumin-to-creatinine ratio, urinary IL-18, kidney injury molecule-1 (KIM-1), TIMP2, IGFBP-7, and uromodulin), with that of FST for the prediction of several clinical end points, including progressive AKI, need for RRT (renal replacement therapy), and inpatient mortality in the same previous published cohort of 77 patients [136]. FST was the best-tested parameter in predicting progression of AKI to stage 3, with an area under the curve (AUC) of 0.87 ± 0.09 (*p* <0.0001). Additionally, FST urine output was the only biomarker to significantly predict RRT (0.86 ± 0.08; *p* = 0.001). Finally, combining FST urine output with individual biomarkers using logistic regression did not significantly improve risk stratification (ΔAUC, *P* >0.10 for all). Plasma NGAL, which performed the best of all the novel biomarkers, had an AUC of 0.86 when pooled with the FST. In this context, it seems that FST is the best available diagnostic tool in predicting the severity of AKI and the need for RRT [136].

In the last year, several renal protective strategies have been attempted [137], unfortunately with poor results [138]. In a large study including 820 patients receiving elective cardiac surgery, there were no favorable effects of statin administration on AKI incidence. Following cardiac surgery and cardiopulmonary bypass renal ischemia-reperfusion injury, impaired vasodilatation, neurohormonal activation, oxidative stress, inflammation, and atheroembolism may promote AKI [139]. Statins administration might reduce oxidative stress, improve endothelial function and decrease inflammation [140, 141]. Moreover, in experimental models of ischemia-reperfusion, short-term statin treatment decreases renal dysfunction and inflammation [142, 143]. Based on these data, the authors administrated high dose atorvastatin a day before surgery and continued until hospital discharge in statin naive patients; in those using statins prior to the study, treatment was administered until the day after surgery (https://www.asn-online.org/education/kidneyweek/2015/KW15_Late-Breakers.pdf). There was no difference in the rate of AKI between atorvastatin users and placebo (20.8 % *vs* 19.5 %; *p* = 0.75). In fact, in statin treatment-naïve patients with previous CKD, the rates of AKI were 52.9 % *vs* 15.8 % for treatment versus placebo, respectively (*n* = 36; *p* = .03). In this context, the study was stopped on the recommendation of the U.S. Data and Safety Monitoring Board.

Equally, steroid administration failed to reduce AKI incidence in patients undergoing cardiopulmonary bypass surgery. It is well known that cardiopulmonary bypass determines a systemic inflammatory response syndrome, which activates several inflammatory cytokines, increases endothelial permeability and postoperative mortality and morbidity [144]. Steroids could suppress this important inflammatory process. 7826 patients from 80 hospital or cardiac surgery centers in 18 countries undergoing cardiac surgery with the use of cardiopulmonary bypass were randomized to receive either methylprednisolone (250 mg at anesthetic induction and 250 mg at initiation of cardiopulmonary bypass) or placebo. Methylprednisolone, compared with placebo, did not reduce the risk of death at 30 days (154 [4 %] *vs* 177 [5 %] patients; relative risk (RR) 0.87, 95 % CI 0·70–1·07, *p* = 0.19) or the risk of death or major morbidity (909 [24 %] *vs* 885 [24 %]; RR 1·03, 95 % CI 0·95–1·11, *p* = 0.52). Additionally, the risk of AKI was similar in both groups: (at 14 days: > 50 % increase in creatinine was: 40.9 % in steroid group versus 39.5 % in placebo; at 30 days –acute dialysis 2.6 % in metilprednisolone group versus 2.4 % in placebo). Moreover, because of the adverse-effect profile of the dose of steroids used in this trial, the investigators make the grade 1 recommendation that steroids should not be used in this setting [145].

In patients with mild deterioration of renal function undergoing cardiac catheterization, it is unclear if holding ACEI or ARB reduces contrast-induced AKI. In this circumstances, 208 patients were randomize to hold or to continue ACEI or ARB prior to coronary angiography; the primary outcome was the incidence of AKI defined as an absolute rise in serum creatinine of ≥0.5 mg/dL from baseline and/or a relative rise in serum creatinine of ≥25 % compared with baseline measured at 48–96 h postcardiac catheterization [146]. There was no difference in the primary outcomes between the groups. However, there was a lower rise in mean serum creatinine after the procedure in patients who held ACEI/ARB (0.3 ± 0.5 *vs* 0.1 ± 0.3 mg/dL, *p* = .03). Moreover, the clinical composite of death, myocardial infarction, ischemic stroke, congestive heart failure, rehospitalization for cardiovascular cause, or need for dialysis pre-procedure occurred in 3.9 % who continued ACEI/ARB compared with 0 % who held the ACEI/ARB (HR 0.11, 95 % CI 0.01–2.96, *p* = 0.06) [146].

## Electrolyte disturbance

At the end of the last year, two new products were used to correct hyperkaliemia in renal cohort and general population. Sodium zirconium cyclosilicate (ZS-9), unlike traditional organic polymers such as Kayexalate and the investigational agent patiromer, was engineered with a highly selective, high-capacity inorganic crystalline lattice structure that preferentially entraps monovalent cations (specifically K+ and ammonium (NH4+)) over divalent cations (e.g., Ca2+ and Mg2+) in the gastrointestinal tract. Recent favorable data are provided from the HARMONIZE, a phase 3, multicenter, randomized, double-blind, placebo-controlled trial. More than half of all patients studied had stage 3 chronic kidney disease (estimated glomerular filtration rate <61 mL/min/1.73 m^2^) or diabetes, and nearly 60 % or more were on at least one renin-angiotensin-aldosterone–modifying medication. Finally, all patients had to be asymptomatic (without an identifiable cardiac arrhythmia) in order to be included in the study. The patients (*n* = 258) received 10 g of zirconium cyclosilicate three times daily in the initial 48-hour open-label phase had a significant decline in serum potassium level; median time to normalization was 2.2 h, with 84 % of patients (95 % CI, 79–88 %) achieving normokalemia by 24 h and 98 % (95 % CI, 96–99 %) by 48 h. In the next randomized phase, the largest dose of ZS-9 (15 g/day for 28 consecutive days) resulted in the lowest plasma potassium levels (from 5.55 to 4.4 mEq/L). In this short-term study, adverse events were comparable between zirconium cyclosilicate and placebo [147].

## Cardiovascular diseases in CKD

Left ventricular hypertrophy (LVH) is highly prevalent in CKD patients [148]. In an observational study including 2410 patients form the Coronary Artery Risk Development in Young Adults (CARDIA) cohort, left ventricular mass index was higher in patients with lower eGFRcys even after adjusting for potential confounders [149]. Compared with eGFRcys >90 mL/min/1.73 m2 (*n* = 2,228), eGFRcys of 60 to 75 mL/min/1.73 m2 (*n* = 29) was associated with 5.63 (95 % CI, 0.90–10.36) g/m2.7 greater LVMI (*p* = 0.02). Moreover, every 1 % decline in eGFR was associated with a 0.40 g/m2.7 increase in LVMI after 10 years, but without reaching statistical significance [149]. Additionally, in patients with rapid decline in eGFR (379 patients) a statistically higher LVMI was noted (β coefficient, 1.48; 95 % CI, 0.11–2.83; *p* = 0.03) compared with participants without a rapid eGFRcys decline after adjustment for confounders. The potential mechanisms for this remain speculative and may include alterations in the renin-angiotensin-aldosterone system or vitamin D-PTH-fibroblast growth factor 23 axis (http://ajkdblog.org/2015/09/24).

## Hypertension

Hypertension is the leading risk factor for death and disability-adjusted life-years lost during 2010 (http://www.who.int/healthinfo/global_burden_disease/GlobalHealthRisks_report_full.pdf). Additionally, inter-arm differences of BP are associated with increased cardiovascular and all-cause mortality [150]; this association was showed in several cohorts, with hypertension, diabetes or established cerebrovascular disease but also in general population [151, 152]. Similar data was confirmed also in CKD cohort. In a prospective cohort study of patients with CKD, the increased interarm systolic blood pressure difference ≥10 mmHg was found to be an independent predictor of CV events after a follow-up period of 19 ± 5 months (HR, 1.802, 95 % CI (1.054–3.079); *p* = 0.031) [153]. This association remained significant even after adjustment for classic risk factors, such as history of CV disease [153].

## Anemia

The main reasons for anemia in CKD are reduced erythropoietin production and iron deficiency [122]. It is well demonstrated that erythropoietin administration is associated with hyper viscosity and thrombogenity, and consequently with increased CV and all-cause morbidity and mortality [154]. Iron administration, as an addition to erythropoietin therapy has developed into usual treatment for renal anemia. On the other hand, there are concerning data regarding the safety of this therapy; almost half of the RCT of oral or iv iron therapy reported adverse effects, increased oxidative stress, endothelial damage or kidney injury [155, 156]. Recent risk/benefit analysis of iron supplementation in patients with CKD not on dialysis was finished. Thirty one thousand nine hundred seventy-one adult patients from Taiwan National Health Insurance Research Database with serum creatinine >6 mg/dL and a haematocrit <28 %, treated already with ESA, were further divided into two groups with or without iron supplementation within 90 days after starting ESA therapy [157]. Despite poor enteric absorption and uncertain efficacy for erythropoiesis by oral iron supplementation, only 17.2 % of iron users were treated via IV route administration; the greater part of the patients were treated with oral iron. This large-scale cohort study showed for the first time that oral iron as well as lower dose IV iron (<200 mg/month) supplementation were associated with significant reduced risk of death among predialysis CKD patients (HR 0.85; 95 % CI 0.80–0.90). Additionally, a lower risk of hospitalizations (HR, 0.97; 95 % CI, 0.94–0.99) was founded [157]. This benefit was not showed in patients who were treated with monthly IV iron >200 m. In contrast, a higher risk of faster progression to end-stage renal disease (HR, 1.05; 95 % CI, 1.01–1.08) was described. This association is still a subject of debate. The authors explained that iron supplementation decreased the competing risk of death; the survivors had more exposure time to pre-ESRD milieu and an increased risk for progression to ESRD and initiation of renal replacement therapy. Additionally, it was already showed that higher transferrin saturation (TSAT) is associated with faster CKD progression in non-dialysis CKD [158]. Moreover, parenteral iron induces lipid peroxidation and cell injury in isolated mouse and human tubular epithelial cells [159] and three observational studies showed that short-term IV iron administration caused transient proteinuria and urinary excretion of tubular enzymes [156, 160, 161].

Most frequently, intravenous iron is recommended for CKD patients, due to poor absorption of oral iron in this category of patients. In hemodialysis population there are several studies showing the benefit of intravenous iron for replenishing iron stores, improving anemia, and reducing ESA dosage requirements compared with oral administration [162]. Different data are recently published for CKD patients. Agarwal et al. randomized 136 patients with anemia and iron deficiency and CKD not on dialysis to either oral ferrous sulfate (69 patients to 325 mg three times daily for 8 weeks) or intravenous iron sucrose (67 patients to 200 mg every 2 weeks, total 1 g). The primary outcome was the between-group difference in slope of measured glomerular filtration rate (mGFR) change over 2 years. The study was stopped earlier because a higher risk of serious cardiovascular events and hospitalization for infection was noted in the group with iron intravenous. There were 36 serious cardiovascular events among 19 participants assigned to the oral iron treatment group and 55 events among 17 participants of the intravenous iron group (adjusted incidence rate ratio 2.51 (1.56–4.04)). Infections resulting in hospitalizations had a significant adjusted incidence rate ratio of 2.12 (1.24–3.64). There was a twofold increase in hospitalizations for heart failure, about a fourfold increase in hospitalizations for pneumonia, and a more than threefold increase in risk for skin infections requiring antibiotics in the IV vs the oral iron group. And, although not quite statistically significant, there was a more than 1.22-fold increase for sepsis (*p* = .056) in the IV iron group. The mGFR slope was almost the same in both groups (oral −3.6 ml/min per 1.73 m^2^, intravenous −4.0 ml/min per 1.73 m^2^, between-group difference −0.35 ml/min per 1.73 m2; 95 % confidence interval −2.9 to 2.3) [163].

There are also novel data in dialysis patients regarding an innovative iron compound. Ferric pyrophosphate citrate (FPC, Triferic™) is a carbohydrate-free, water-soluble, complex iron salt that was first demonstrated to deliver iron via dialysate in 1999, allowing maintenance of hemoglobin (Hgb) concentration and iron balance while reducing the need for IV iron by about 80 % [164]. Fishbane et al. showed data from two identical phase 3 RCT (CRUISE 1 and 2), conducted in 599 iron-replete chronic HD patients [165]. Patients were dialyzed with dialysate containing 2 μM FPC-iron or standard dialysate (placebo) for up to 48 weeks. Oral or intravenous iron supplementation was prohibited, and doses of erythropoiesis-stimulating agents were held constant. Hemoglobin concentration was maintained constant in the FPC group but decreased by 0.4 g/dL in the placebo group (*p* <0.001, combined results; 95 % CI 0.2–0.6). Placebo treatment resulted in significantly higher decreases from baseline in reticulocyte hemoglobin content (−0.9 pg versus −0.4 pg, *p* <0.001) and serum ferritin (−133.1 μg/L versus −69.7 μg/L, *p* <0.001) compared with FPC treatment [165]. Additional data were provided by the PRIME study. This 9-month, randomized, placebo-controlled, double blind, multicenter clinical study included 103 patients undergoing HD. The FPC group received dialysate containing 2 μmol/l of iron. The placebo group received standard dialysate. Intravenous iron was administered according to the approved indication when ferritin levels fell below 200 μg/l. ESA use was 35 % lower in dialysate iron group compared with placebo. Interestingly, ESA use increased in both groups, but much more in the placebo group. Fewer patients in the dialysate iron group needed or received IV iron (51 % less iv iron). Adverse and serious adverse events were similar in both groups [166].

## Dialysis

Mortality risk in hemodialysis patients remains elevated and is mostly linked with cardiovascular complication and infection [167, 168]. A better strategy to recognize those patients with increase risk of death is desirable. Recently, a new model was validated in the second Analyzing Data, Recognizing Excellence and Optimizing Outcomes (AROii) cohort. This database of 11,508 entries contains demographic and biochemical observations of incident hemodialysis patients (median dialysis vintage, 4 months). This scoring model was validated externally using similar-sized Dialysis Outcomes and Practice Patterns Survey (DOPPS) data [169]. For AROii, the observed 1- and 2-year mortality rates were 13.0 (95 % confidence interval (CI; 12.3–13.8) and 11.2 (10.4–12.1)/100 patient years, respectively. The greatest predictor of mortality was patient age, with the risk for mortality beginning at age 60 and increasing thereafter. C-reactive protein and albumin were the best biochemical parameters that contributed to mortality. Additionally, low body mass index, history of cardiovascular disease or cancer, and use of a vascular access catheter during baseline were consistent predictors of mortality [169].

The benefit of dialysis is undeniably extended survival in patients with ESRD. However, elderly patients on dialysis have a high incidence of chronic health conditions, frailty, falls and cognitive impairment [170]. In this context, life expectancy for many elderly ESRD patients is similar or worse than that associated with common cancers, and HD does not always substantially prolong life among older adult [171]. A shared decision-making tool could help elderly advanced CKD patients decide about initiating dialysis. Recently, a predictive risk score for early mortality after dialysis initiation in the elderly was derived and validated. Using US Renal Data System and claims data from the Centers for Medicare & Medicaid Services for 69,441 (aged ≥67 years) patients with end-stage renal disease who initiated dialysis therapy from January 1, 2009, to December 31, 2010 a simple risk score was validated. This simple risk score (total score, 0–9) included age (0–3 points), low albumin level, assistance with daily living, nursing home residence, cancer, heart failure, and hospitalization (one point each); The highest scores (above eight) indicated an estimated 39 and 55 % probability of death within the first 3 and 6 months, respectively [172]. For a patient with a score of three, the estimated probability of death at 3 and 6 months were 12 and 20 %, respectively [172].

Hemodialysis is the most routinely used renal replacement therapy for end stage renal disease (ESRD) patients. Arterio-venous fistulae remain the most prevalent access for hemodialysis. The rates of thrombosis and maturation failure are still high; at 1 year primary patency rates vary between 50 and 80 % [173]. Fish oil with its anti-proliferative, anti-oxidant, and vasodilator effects, could theoretical be efficient for preventing development of AV-graft stenosis and thrombosis [173]. However, in a randomized control trial, neither aspirin or fish oil administration did not improve the FAV patency at 1 years in adult patients with CKD stage 4 or 5, already on HD on where HD is planned to start within 6 months [174]. Four grams of fish oil or placebo were given for 12 weeks to 567 patients and a subgroup of patients were assigned to aspirin 100 mg or placebo. There was an overall rate of 47 % AVF failure and aspirin or fish oil did not change the outcomes [174].

Uremic pruritus affects almost 50 % of patients with ESRD and often causes sleep disturbance, long term pain and impaired quality of life [175]. Recent presented data showed that nalbuphine, an opioid agonist reduces symptoms of uremic pruritus [176]. Three hundred seventy-three hemodialysis patients were assigned to take nalbuphine 120 mg (*n* = 120), nalbuphine 60 mg (*n* = 128) or placebo (*n* = 125) for a period of 6 weeks [177]. Primary outcome was the numerical rating scale score (NRS) of worst itching intensity for each dose of nalbuphine versus placebo. Patients receiving 120 mg nalbuphine showed a significant reduction of itch intensity by 49 %. A non-significant reduction was also noted in the 60 mg group; a post-hoc analysis showed that patients who received 120 mg and had at baseline a NRS score of seven or above had not only a significant NRS reduction, but also an improvement in sleep quality according to the ITCH Medical outcomes Study sleep scale. Most adverse events were nausea and vomiting; they were not different between dose treatment groups and were attenuated after the second week of treatment [177].

## Conclusions

Year 2015 has been extremely important and interesting in the field of lipid, blood pressure and kidney research. IMPROVE-IT trial revalidated the concept “lower is better” for LDL-C as a target for therapy, increasing the necessity of treatment the high-risk patients to achieve LDL-C goals, and suggesting the benefits of new goal of 50 mg/dL and lower. For the statin-intolerant patients and those requiring essential reductions in LDL-C to achieve their goals, new therapies, including PCSK9 inhibitors remain promising drugs. In BP research, AHA/ACC 2015 guidelines recommended a target for BP below 140/90 mmHg in stable or unstable CAD patients and below 150/90 mmHg in patients older than 80 years of age, however the recent results of the SPRINT trial have suggested that there might be significant benefits, taking into account CV risk, for hypertensive patients over 50 without diabetes and BP levels <120/80. Finally, in kidney research, reducing the progression of CKD and related complications such as anemia, metabolic acidosis, bone and mineral diseases, AKI and CVD is still a goal for clinicians.
